# Lateral pharyngeal wall myeloid sarcoma as a relapse of acute biphenotypic leukemia: a case report and review of the literature

**DOI:** 10.1186/1752-1947-7-292

**Published:** 2013-12-30

**Authors:** Redha Alrumaih, Muhammad Saleem, Suresh Velagapudi, Mohammad Anas Dababo

**Affiliations:** 1 Department of Otolaryngology/Head and Neck Surgery and Communication Sciences, King Faisal Specialist Hospital and Research Centre, Riyadh, Saudi Arabia; 2 Department of Pathology and Laboratory Medicine, King Faisal Specialist Hospital and Research Centre, Riyadh, Saudi Arabia

**Keywords:** Acute biphenotypic leukemia, Extramedullary tumor, Lateral pharyngeal wall, Myeloid sarcoma

## Abstract

**Introduction:**

Myeloid sarcoma is a rare extramedullary malignant tumor composed of immature myeloid cells. The tumor can affect any part of the body. Involvement of the oral cavity and nasopharynx has been reported in 50 cases. We report a case describing myeloid sarcoma affecting the lateral pharyngeal wall.

**Case presentation:**

A 31-year-old Arabian man who had acute biphenotypic leukemia treated with chemoradiation and allogeneic stem cell transplant was referred to our department with sore throat and a mass lesion in his lateral pharyngeal wall after failed antibiotic therapy. Biopsy of his lesion revealed myeloid sarcoma. He was referred to the Department of Hematology-Oncology for further evaluation that showed no other lesions.

The patient was diagnosed with isolated extramedullary myeloid sarcoma of his lateral pharyngeal wall as a relapse of acute biphenotypic leukemia and managed with chemoradiation.

**Conclusions:**

Myeloid sarcoma of the pharynx is a rarely encountered malignancy in the practice of otolaryngology-head and neck surgery. It can develop *de novo*, but may also represent relapse of leukemia. Thus, it should be considered in the differential diagnosis of any pharyngeal lesions in patients with leukemia.

## Introduction

Myeloid sarcoma (MS) is a rare extramedullary malignant tumor composed of immature myeloid cells. Burns first described the lesion in 1811 [1] and King first used the term chloroma in 1853 because the tumor often exhibits a greenish color on exposure to air, owing to the presence of myeloperoxidase in the tumor cells [2]. Dock described the relationship between acute myeloid leukemia (AML) and granulocytic sarcoma in 1893 [3]. Rappaport used the term “granulocytic sarcoma” because microscopically the tumor is composed of immature granulocytic cells and resembles a sarcoma [4].

MS is often discovered concomitantly or subsequently to previously diagnosed AML. It can also be found as an isolated lesion or may precede the disease in the bone marrow [5]. We present a case of a 31-year-old Arabian man who presented with a lateral pharyngeal wall mass diagnosed as an isolated extramedullary MS and as a relapse of acute biphenotypic leukemia 4 years after allogeneic stem cell transplant (ASCT).

## Case presentation

A 31-year-old Arabian man, diagnosed with acute biphenotypic leukemia and treated with chemoradiation followed by ASCT 4 years previously, was referred from the Department of Hematology-Oncology. He had a 1-month history of sore throat not responding to two courses of oral antibiotics. His sore throat was not associated with fever, upper respiratory tract infection symptoms, chills, rigor, fatigue or weight loss. His medical history was significant for recently diagnosed and controlled essential hypertension, treated hepatitis B infection, and treated mucormycosis of the mandible encountered during the course of ASCT.

**Figure 1:**
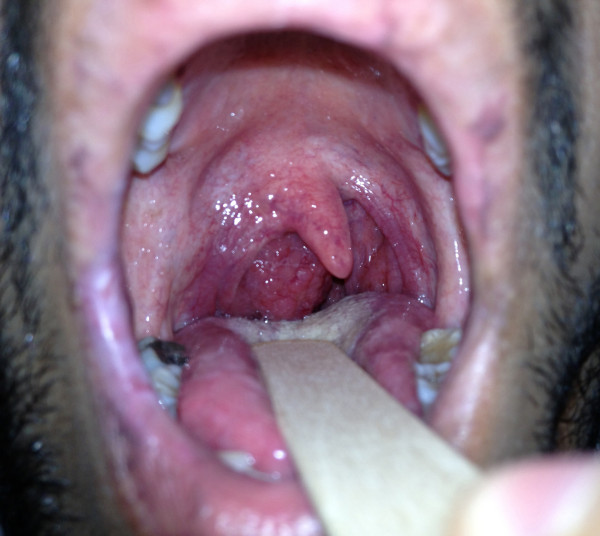
Clinical photo showing granular mass arising from the right lateral pharyngeal wall and pushing the uvula to the contralateral side.

On examination, he was in good general health. An oropharyngeal examination revealed a 3×2cm granular mass in his right lateral pharyngeal wall reaching the midline and pushing the uvula to the contralateral side (Figure 1). It resembled a unilateral tonsillar enlargement with a normal contralateral tonsil. The rest of the ears, nose and throat examination, including examination of his post-nasal space and larynx, did not reveal any abnormality. There was no cervical or systemic lymphadenopathy. The systemic examination was unremarkable.

**Figure 2:**
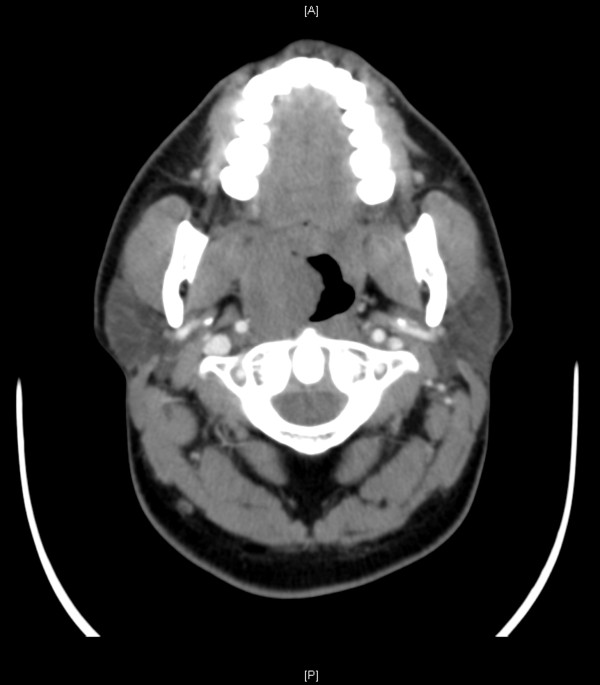
Iodinated, contrast-enhanced computed tomography scan of the head and neck (cross-section) showing non-enhancing, homogenous enlargement of the right tonsil.

A complete blood count showed normal hemoglobin level and normal platelet and white cell counts. The coagulation profile, liver enzymes and electrolytes were all within normal limits. An iodinated, contrast-enhanced computed tomography (CT) scan of his head and neck showed right tonsillar enlargement (2.7×2.3cm; Figure 2). Apart from this, the CT scan did not show any other abnormalities or localized collections. There was no sign of enlarged lymph nodes.

In view of the history of leukemia, a tonsillectomy was carried out after taking informed consent. Intraoperatively, the mass was found to arise from the right lateral pharyngeal wall, obscuring a normal-appearing right tonsil. There was no surgical plane between the mass and the lateral pharyngeal wall. Because of the friability of the mass, the majority of the tumor was removed in a piecemeal fashion. Bleeding was minimal. Postoperative recovery was uneventful.

**Figure 3:**
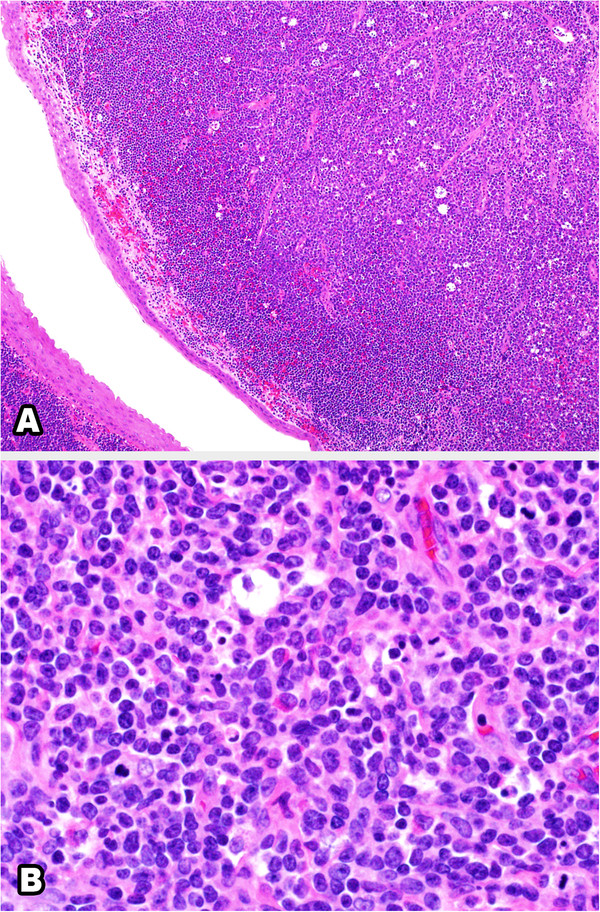
**Hematoxylin and eosin stained sections of the lesion. (A)** Low-power section showing subepithelial diffuse infiltrate of small blue cells with starry sky appearance. **(B)** High-power section showing myeloblasts with evident eosinophil precursors.

**Figure 4:**
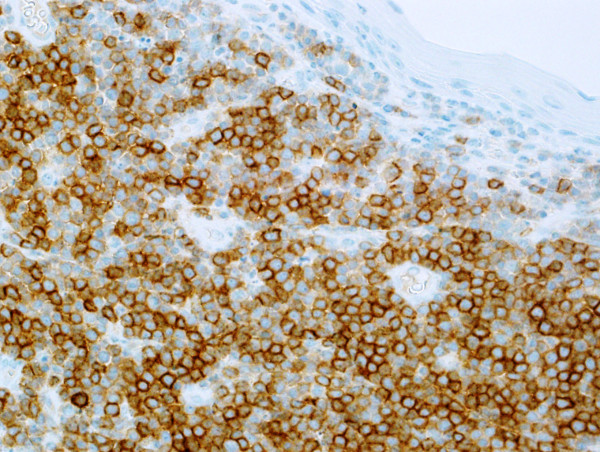
CD117.

**Figure 5:**
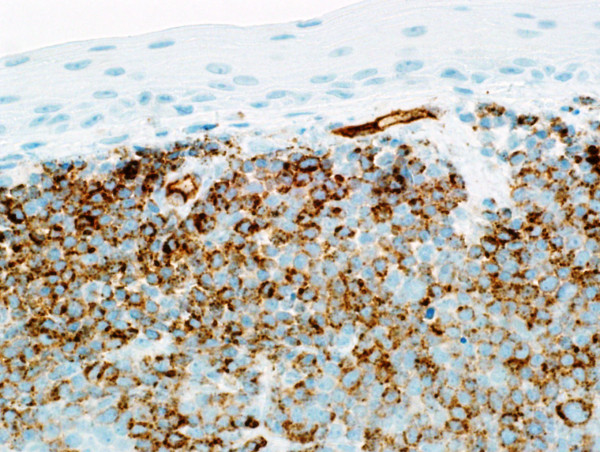
CD34.

**Figure 6:**
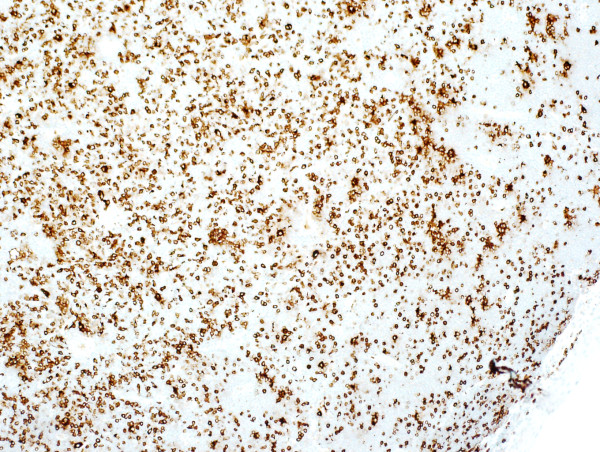
Myeloperoxidase.

Microscopic examination revealed effacement of the subepithelial tissue by infiltrating small, round blue cells, with some starry sky appearance in the background due to scattered large foamy macrophages (Figure 3A). The infiltrating neoplasm rarely shows early differentiation with evident eosinophilic precursors (Figure 3B). The tumor cells were positive for CD117 (Figure 4), CD34 (Figure 5), myeloperoxidase (Figure 6) and lysozyme, while negative for CD20, CD3, terminal deoxynucleotidyl transferase, CD99 and cytokeratin. A portion of the fresh tissue was also sent for flow cytometric evaluation, which showed a dominant myeloid phenotype and positivity for CD45, CD34 (dim), CD117, CD33, CD13 (dim), CD7, CD38 and human leukocyte antigen-DR. T-lymphoid-associated or B-cell antigens were not detected.

The patient was referred back to the Hematology-Oncology Department for further evaluation and definitive treatment of MS. Further work-up, including diagnostic lumbar puncture and bone marrow biopsy, were negative for leukemia. A gadolinium-enhanced magnetic resonance imaging scan of his brain and spine as well as an abdominal ultrasound found no abnormalities consistent with disseminated disease. No clonal chromosomal abnormalities were found from a chromosomal analysis.

**Figure 7:**
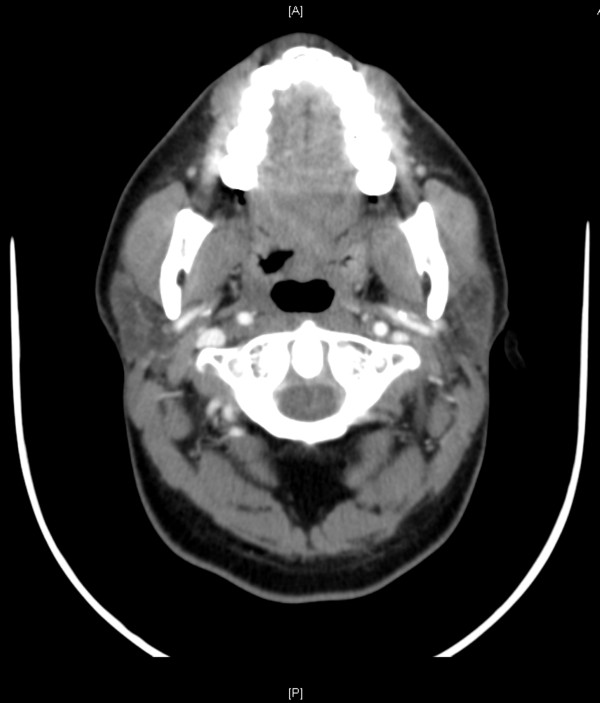
Computed tomography scan of the head and neck (cross-section) after chemoradiation showing resolution of the lesion.

We made the diagnosis of an isolated extramedullary MS of the lateral pharyngeal wall as a relapse of acute biphenotypic leukemia. The case was discussed at the departmental interdisciplinary tumor board and the decision was made to treat the patient with chemoradiation. He received five cycles of chemotherapy using cytarabine and fludarabine as well as radiation treatment using 1500cGy in five fractions to his throat. A CT scan of his head and neck 1 month after completion of chemoradiation showed complete resolution of the pharyngeal mass (Figure 7). The patient was under the regular surveillance protocol by the Departments of Hematology-Oncology and Otolaryngology at the time of writing.

## Discussion

MS is a well-known entity to the hematologist. However, it is rarely encountered in otolaryngology practice. The tumor can affect any part of the body. The commonly involved sites include subperiosteal bone, lymph nodes and skin [6]. The head and neck region is affected in 12% to 43% of cases [7]. Oral involvement is rare and only 37 cases have been described between 1883 and 2008 [8]. We found an additional 10 cases in the English literature from 2009 to March 2013 (Table 1). The palate, tongue, upper and lower alveolus, buccal mucosa, lips, tonsils and periapical region have all been affected. In addition, we found three cases affecting the nasopharynx and our case describes MS affecting the lateral pharyngeal wall (Table 2).

**Table 1:** Summary of reported cases of intraoral myeloid sarcoma from 2009 to March 2013

**Authors**	**Year**	**Patient age (year)/Sex**	**Type of malignancy**	**Location**
King Kim *et al*. [9]	2009	4/F	AML*	Mandible
Tuntiwong and Kiatmanakul [10]	2009	16/F	*De novo*	Mandible
Cheng *et al*. [11]	2009	56/M	AML relapse	Buccal mucosa
Osterne *et al*. [8]	2009	23/F	AML relapse	Mandible
da Silva-Santos *et al*. [12]	2010	47/F	CML relapse	Gingiva
Fasanmade *et al*. [13]	2010	75/F	MDS	Mandible
Papamanthos *et al*. [14]	2010	72/F	AML	Mandible
Cheng *et al*. [15]	2011	57/M	MDS	Tonsil
Dym and Movahed [16]	2011	16/F	AML relapse	Palate
Seema *et al*. [17]	2011	5/M	*De novo*	Mandible

*Abbreviations*: *AML* acute myeloid leukemia, *CML* chronic myeloid leukemia, *F* Female, *M* Male, *MDS* myelodysplastic disorders; *Myeloid sarcoma was the presenting lesion.

**Table 2:** Summary of reported cases of pharyngeal myeloid sarcoma

**Authors**	**Year**	**Patient age (year)/Sex**	**Type of malignancy**	**Location**
Sugimoto *et al*. [18]	2004	31/F	AML relapse	Nasopharynx
Selvarajan *et al*. [19]	2008	25/M	AML relapse	Nasopharynx
Cho *et al*. [20]	2011	18/M	*De novo*	Nasopharynx
Present case	2013	31/M	Biphenotypic leukemia relapse	Lateral pharyngeal wall

*Abbreviations*: *AML* acute myeloid leukemia, *F* Female, *M* Male.

MS can be encountered either as an isolated lesion or during a course of AML, chronic myeloid leukemia, myelodysplastic syndrome or myeloproliferative disorders. It might be detected during the initial diagnosis of these diseases, present during the relapse or be the first sign of relapse itself [5,21,22]. Isolated lesions, occurring in 8% to 20% of cases, appear to be more common in the relapse of ASTC patients [23]. The reason for this association is still unknown, but could be attributed to a pattern of graft-versus-leukemia surveillance or the biology of high-risk AML treated with transplantation [24].

Being a rare disease, the diagnosis of MS can be challenging, especially if not preceded by leukemia or other bone marrow disease. In general, in any patient who presents with a non-infective unilateral pharyngeal wall lesion, malignancy should always be ruled out. In our case, the appearance of a unilateral pharyngeal mass not responding to medical treatment immediately raised the suspicion of malignancy, especially in view of the patient’s hematological history. The differential diagnoses generally include lymphoma, squamous cell carcinoma and relapse of acute leukemia.

On histologic examination, MS shows variable morphology. It can present with infiltration of cells at all stages of myeloid differentiation; hence, it can be easily recognized by the presence of eosinophilic myelocytes. However, it can present with blastic cells with little or no granulocytic differentiation, making it difficult to diagnose by hematoxylin and eosin staining. Thus, it can occasionally be difficult to differentiate MS from extramedullary hematopoiesis or other neoplasms such as lymphoma, undifferentiated small round cell tumor and malignant melanoma. Immunohistochemistry is helpful in such cases [25]. In our case, the presence of eosinophilic precursors was a very helpful feature and the morphology was very suggestive of the diagnosis. This was confirmed by immunohistochemistry and flow cytometry.

Because of the lack of randomized prospective studies, there is no consensus on the treatment of MS. The current recommended treatment regimen is similar to the conventional AML chemotherapeutic protocols. The role of concomitant radiotherapy is not established although it is often given [26-28].

Owing to the rarity of MS, randomized controlled trials to address prognostic factors are difficult to perform. Overall, there seems to be a difference in prognosis between MS patients presenting with an isolated lesion compared with those who have concomitant leukemia or relapse. The latter have a poorer outcome and shorter survival [26]. In terms of specific prognostic factors, Pileri and colleagues analyzed 92 patients with MS and found that disease course and response to therapy were not influenced by patients’ age, gender, anatomic location, clinical presentation, previous clinical history, morphological classification, immunophenotype and cytogenetic findings [25].

## Conclusions

MS of the pharynx is a rarely encountered malignancy in the practice of otolaryngology-head and neck surgery. It can develop *de novo* but may also represent relapse of leukemia. Thus, it should be considered in the differential diagnosis of any pharyngeal lesions in patients with leukemia.

## Consent

Written informed consent was obtained from the patient for publication of this case report and accompanying images. A copy of the written consent is available for review by the Editor-in-Chief of this journal.

## Abbreviations

AML: Acute myeloid leukemia; ASCT: Allogeneic stem cell transplant; CT: Computed tomography; MS: Myeloid sarcoma.

## Competing interests

The authors declared that they have no competing interests.

## Authors’ contributions

RA conceived the idea, wrote the manuscript and was directly involved in the medical care provided to the patient. MS helped in drafting the manuscript and revised it critically for important intellectual content. SV was the primary surgeon and gave final approval of the version of this manuscript to be published. MAD performed the histological examination of the lesion and prepared the histology photographs. All authors read and approved the final manuscript.
